# Deep Sequencing Reveals the Effect of MeJA on Scutellarin Biosynthesis in *Erigeron breviscapus*

**DOI:** 10.1371/journal.pone.0143881

**Published:** 2015-12-14

**Authors:** Rui-Bing Chen, Jiang-Hua Liu, Ying Xiao, Feng Zhang, Jun-feng Chen, Qian Ji, He-Xin Tan, Xin Huang, Hao Feng, Bao-Kang Huang, Wan-Sheng Chen, Lei Zhang

**Affiliations:** 1 Department of Pharmaceutical Botany, School of Pharmacy, Second Military Medical University, Shanghai 200433, P. R. China; 2 School of Forestry, Southwest Forestry University, Kunming, Yunnan, 650224, P. R. China; 3 Department of Pharmacy, Shanghai Changzheng Hospital, Second Military Medical University, Shanghai 200003, P. R. China; 4 Business Development & Strategic Planning Central Research Institute, Shanghai Pharmaceuticals Holding Co., ltd, Shanghai 200021, P. R. China; 5 Department of Pharmacognosy, School of Pharmacy, Second Military Medical University, Shanghai 200433, P. R. China; Chinese Academy of Medical Sciences, Peking Union Medical College, CHINA

## Abstract

**Background:**

*Erigeron breviscapus*, a well-known traditional Chinese medicinal herb, is broadly used in the treatment of cerebrovascular disease. Scutellarin, a kind of flavonoids, is considered as the material base of the pharmaceutical activities in *E*. *breviscapus*. The stable and high content of scutellarin is critical for the quality and efficiency of *E*. *breviscapus* in the clinical use. Therefore, understanding the molecular mechanism of scutellarin biosynthesis is crucial for metabolic engineering to increase the content of the active compound. However, there is virtually no study available yet concerning the genetic research of scutellarin biosynthesis in *E*. *breviscapus*.

**Results:**

Using Illumina sequencing technology, we obtained over three billion bases of high-quality sequence data and conducted *de novo* assembly and annotation without prior genome information. A total of 182,527 unigenes (mean length = 738 bp) were found. 63,059 unigenes were functionally annotated with a cut-off E-value of 10^−5^. Next, a total of 238 (200 up-regulated and 38 down-regulated genes) and 513 (375 up-regulated and 138 down-regulated genes) differentially expressed genes were identified at different time points after methyl jasmonate (MeJA) treatment, which fell into categories of ‘metabolic process’ and ‘cellular process’ using GO database, suggesting that MeJA-induced activities of signal pathway in plant mainly led to re-programming of metabolism and cell activity. In addition, 13 predicted genes that might participate in the metabolism of flavonoids were found by two co-expression analyses in *E*. *breviscapus*.

**Conclusions:**

Our study is the first to provide a transcriptome sequence resource for *E*. *breviscapus* plants after MeJA treatment and it reveals transcriptome re-programming upon elicitation. As the result, several putative unknown genes involved in the metabolism of flavonoids were predicted. These data provide a valuable resource for the genetic and genomic studies of special flavonoids metabolism and further metabolic engineering in *E*. *breviscapus*.

## Introduction

*Erigeron breviscapus* (Vant.) Hand-Mazz. (Deng Zhanhua in Chinese) is a famous traditional Chinese medicine, broadly used for cerebrovascular disease in clinic [1]. The main active compounds of *E*. *breviscapus* are flavonoids and their derivatives, such as scutellarin and apigenin 7-O-glucuronide [2]. More than 95% of the clinical efficiency *E*. *breviscapus* is performed for the treatment of cerebrovascular disease and apoplexy sequelae [3]. However, such active nature compounds are only found in a few *Erigeron* species, including *E*. *breviscapus* and *E*. *multiradiatus*. What's worse, *E*. *breviscapus*, mainly distributed in Yunnan province in China, is endangered due to overexploitation [4].

Though the biosynthesis of flavonoids in many plants has been well characterized [5–7], only a few key genes including enzyme-encoding genes and transcription factors (TFs) involved in the biosynthesis of scutellarin have been cloned from *E*. *breviscapus* without any function confirmation [8–10]. The biosynthesis of flavonoids origins from phenylpropanoid pathway, and L-phenylalanine is transformed into 4-coumaroyl-CoA by phenylammonia lyase (PAL), cinnamate-4-hydroxylase (C4H) and coumaroyl-CoA-ligase (4CL). And then one 4-coumaroyl-CoA molecule with three malony-CoA molecules produces a naringenin chalcone (4, 2', 4', 6'-tetrahydroxychalcone) through chalcone synthases (CHS). Through chalcone isomerase (CHI) and flavone synthase II (FNS II), chalcone is transformed into apigenin. In some plants, flavonoids 6-hydroxylase (F6H) have been found, which however have been insufficiently studied due to their restricted occurrence. F6H is a cytochrome P450-dependent monooxygenase (CYP71D9) in soybean [11], but a 2-oxoglutarate-dependent dioxygenase (ODD) in *Chrysosplenium americanum* [12]. Furthermore, because there is a 6-OH in the scutellarin structure, we believe there must be a *F6H* that converts apigenin to scutellarein in *E*. *breviscapus*, like F6Hs in other plants abovementioned. Meanwhile, FNS II and F6H may be in no particular order. Finally, scutellarein is glycosylated by flavonoid 7-O-glucuronosyltransferase (F7GAT) to scutellarin and F7GAT is firstly purified in *S*. *baicalensis* [12].

TFs are proteins that bind to specific DNA sequences in the promoters to control and regulate the transcription of genes. Many TF families, such as WRKY, MYB and bHLH, perform extensive regulatory effects to the biosynthesis of flavonoids in plant kingdom [13]. In grape, *VvMYB5b* can activate the transcription of *ANS*, *CHI* and *LAR1* directly and contribute to the regulation of anthocyanin and proanthocyanidin biosynthesis [14]. In most cases, MYB-bHLH-WD40 always participates in the biosynthesis of flavonoids as a protein complex [15]. In rice, *OsWRKY13* has been found to show effects on the biosynthesis of flavonoids [16]. However, it is still unknown as how TFs regulate the biosynthesis of scutellarin in *E*. *breviscapus*.

Many signal molecules, including Methyl jasmonates (MeJA), abscisic acid (ABA) and salicylic acid (SA) show extensive regulations to the secondary metabolism containing biosynthesis of flavonoids [17]. On the one hand, in our previous research, the yields of rosmarinic acid and tanshinone in *Salvia miltiorrhiza* and lignans in *Isatis indigotica* were significantly induced by exogenous MeJA. In addition, it is well known that most MeJA-induced plants show a larger accumulation of flavonoids [18]. On the other hand, analysis after MeJA treatment also can be used to unveil the relation between gene and metabolism, find key genes involved in the biosynthesis of active compounds and conduct metabolic engineering [19, 20]. However, the effect of MeJA on the biosynthesis of scutellarin in *E*. *breviscapus* has not been elucidated up to now.

To get a comprehensive understanding of the global regulation mechanism of MeJA on scutellarin biosynthesis in *E*. *breviscapus*, the transcriptomes of MeJA-induced *E*. *breviscapus* seedlings were performed in this study. Comparison of the gene expression profiles among three independent groups (0 h, 3 h, and 12 h after treatment) revealed the genes that were up- or down-regulated at different time of MeJA treatment and two gene co-expression networks were analyzed to find 13 putative flavonoids genes.

## Result and Discussion

### 2.1 Illumina sequencing and *de novo* assembly of sequence reads in *E*. *breviscapus*

**Fig 1:**
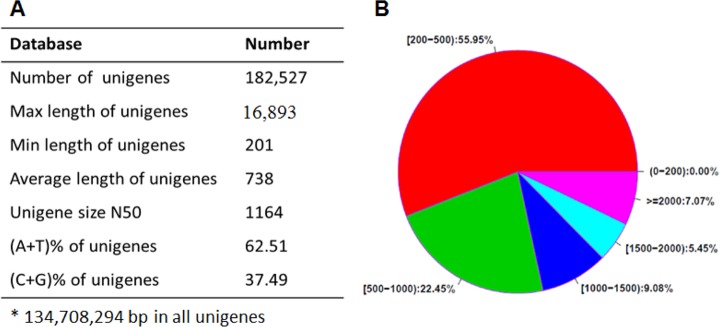
Summary of sequences analysis. A) Basic data of sequences, B) Length distribution of the sequences.

The sequencing produced 5,409,932 reads with a total of 332,496,270 nucleotides (nt). The number of all unigenes was 182,527, and the max and min lengths of unigenes were 16,893 and 201, respectively (Fig 1A). Of these unigenes, the length of about 44.05% was more than 500 bp, which was great for further deep analyses (Fig 1B).

### 2.2 COG and GO classification and KEGG pathway enrichment of unique sequences

**Fig 2:**
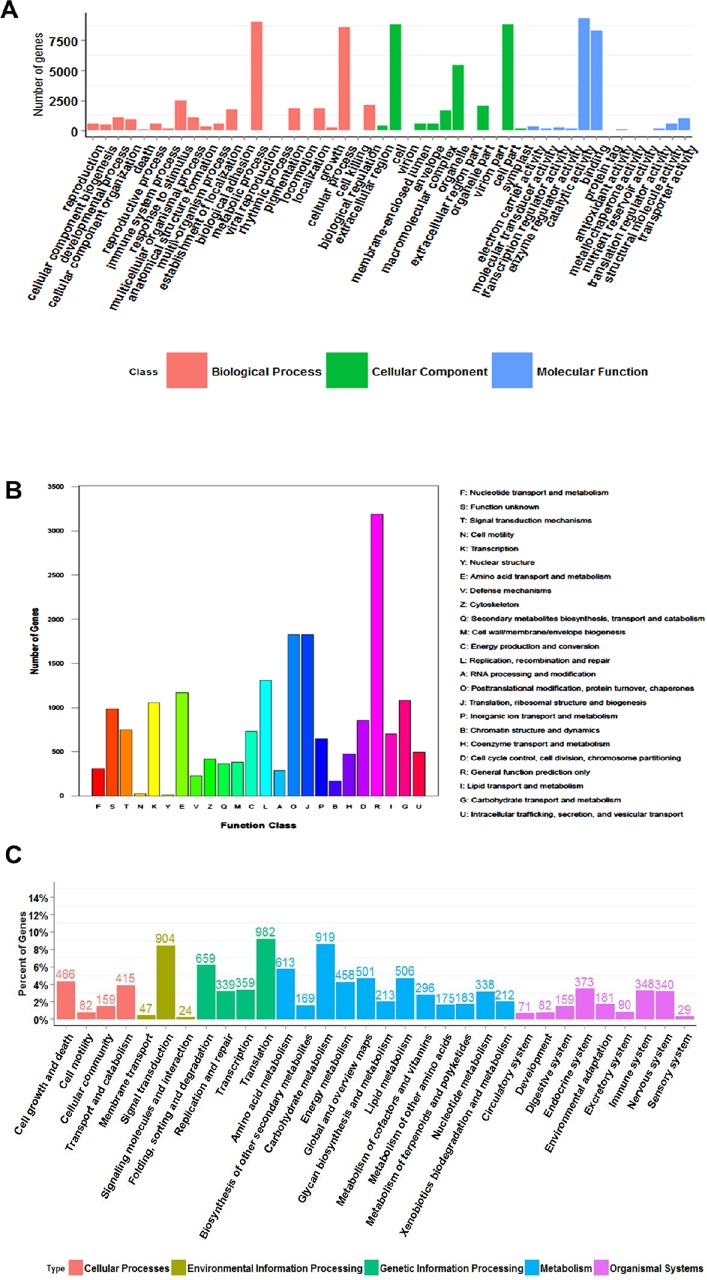
Functional classification of the assembled unigenes. A) Functional classification of the assembled unigenes based on Gene Ontology (GO) categorisation. 17,078 unigenes were summarised in three main GO categories: ‘biological processes’, ‘cellular components’ and ‘molecular functions’. The Y-axis represents the number of genes, and X-axis represents the different categories. B) COG classification of the putative proteins. 19,274 unigenes were aligned to the COG database to predict and classify possible functions and were classified into 24 different functional COG classes. The Y-axis indicates the number of unigenes in a specific functional cluster and the X-axis indicates the symbols of different functional COG classes. C) Histogram presentation of the KEGG classification of the annotated transcripts. A total of 7,984 unique sequences were annotated into 33 pathways of 4 groups (‘Cellular Processes’, ‘Environmental Information Processing’, ‘Metabolism’ and ‘Organismal Systems’). The X-axis indicates the KEGG pathway, and the Y-axis indicates the percentage of unigenes that were assigned to a specific pathway.

The Gene Ontology (GO) analysis was used to classify the functions of all unigenes. A total 17,078 (9.4%) unigenes were classified into 47 subcategories and three main categories. In ‘biological processes’ group, two subgroups, ‘cellular process’ and ‘metabolic process’ had more members than others, indicating that the plants were undergoing extensive metabolic activities. For the ‘cellular components’, the assignments were mainly given to the ‘cell’, ‘cell part’ and ‘organelle’. ‘Binding’ and ‘catalytic active’ were the most highly represented groups under the ‘molecular function’ category (Fig 2A). The statistics abovementioned also accorded with such plants as *Sophora moorcroftiana* [21], *Prosopis alba* [22] and *Chorispora bungeana* [23]. This analysis provided a clue of the gene function, which might help further studies on gene function prediction.

To classify the orthologous gene products, through the Cluster of Orthologous Groups of proteins (COG) database, 19,274 unique (10.6%) sequences were annotated and classified into 24 different functional COG classes, which were represented by A to Z. The largest group was the ‘general function prediction only’, followed by ‘translation, ribosomal structure and biogenesis’; ‘posttranslational modification, protein turnover, chaperones’; and ‘replication, recombination and repair’ (Fig 2B). Based on the COG analysis, this work helped us to better understand the protein function without reference genome sequence and protein evolution.

To further understand the active biochemical pathways in *E*. *breviscapus*, unigenes were mapped into reference canonical pathways in Kyoto Encyclopedia of Genes and Genomes (KEGG) (Fig 2C). A total of 7,984 unique sequences (4.4%) were annotated into 33 pathways of 4 groups (‘Cellular Processes’, ‘Environmental Information Processing’, ‘Metabolism’ and ‘Organismal Systems’). By comparing the GO and COG analyses, the pathway-based analysis may predict the accurate function of genes in metabolism. A summary of the unigenes annotation is given in S1 Table.

### 2.3 Candidate genes encoding enzymes involved in the biosynthesis of scutellarin

**Fig 3:**
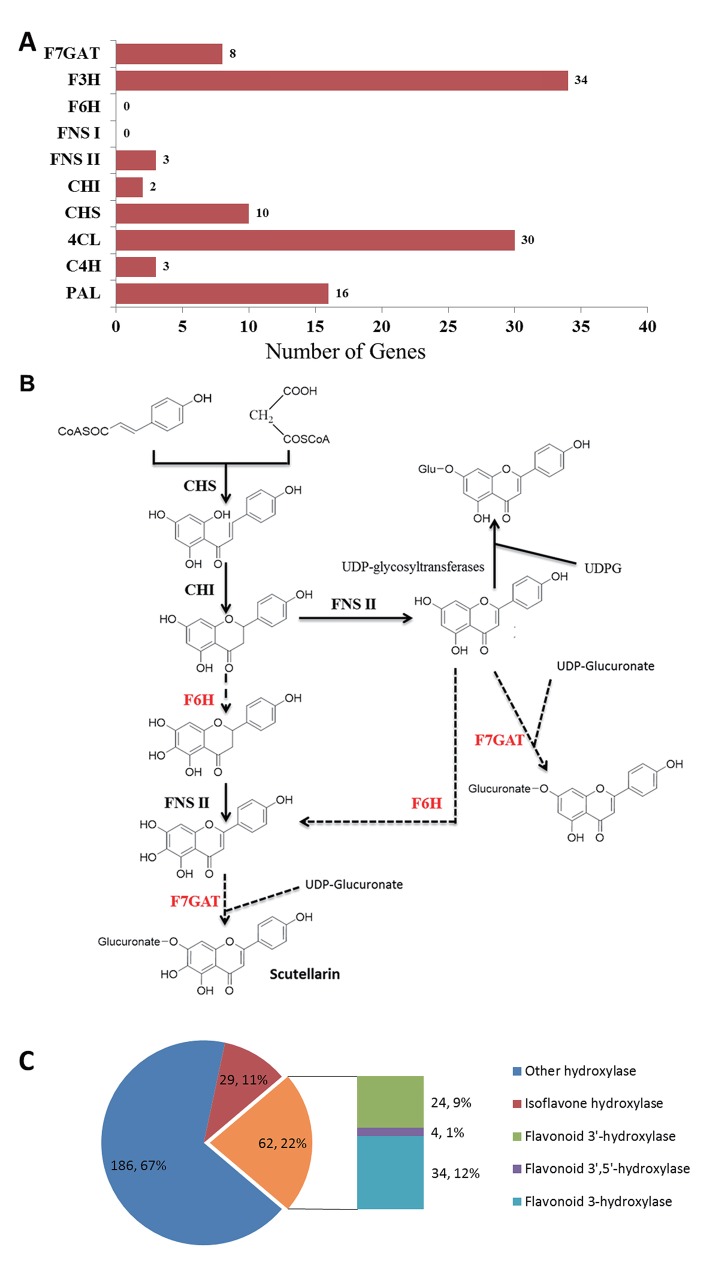
Transcripts involved in flavonoids biosynthesis in *E*. *breviscapus*. A) Identification of the catalyzing enzymes involved in flavonoids biosynthesis. The X-axis indicates the number of genes, and the Y-axis indicates the name of genes. B) Putative biosynthesis of scutellarin in *E*. *breviscapus*. Full and dashed lines mark defined and putative steps in plants, respectively. The red words indicate the unknown steps in plants. Enzyme abbreviations are as follows: CHI: chalcone isomerase; CHS: chalcone synthase; FNS II: Flavone synthase II; F6H: flavonoid 6-hydroxylase; F7GAT: flavonoid 7-O-glucuronosyltransferase. C) All annotated hydroxylases in unigenes using Nr databases. No *F6H* was found.

Based on Nr datasets, the numbers of the unigenes involved in the biosynthesis of scutellarin were discovered in this study (Fig 3A), including *PAL*, *C4H*, *4CL*, *CHS*, *CHI*, *FNS II*, *FNS I*, *F6H*, *F3H*, *UGT*.

We have previously mentioned that there must be a F6H, which converts apigenin to scutellarein, followed by glucuronidation catalyzed by F7GAT (Fig 3B). According to the results, 8 *F7GAT* were identified, which might convert scutellarein to scutellarin. Unfortunately, candidate *F6H* was not found in this result and the same result was found in Yang’ research [4]. Furthermore, all hydroxylases were discovered and classified into three groups (isoflavone hydroxylase, flavonoids hydroxylase and other hydroxylase). The result showed that the flavonoids hydroxylases only included flavonoid 3'-hydroxylases (24, 9%), flavonoid 3', 5'-hydroxylases (4, 1%) and flavonoid 3-hydroxylases (34, 14%). The reason for this result might be that the F6H has been insufficiently studied due to its restricted occurrence (Fig 3C).

### 2.4 Identification of transcription factors

**Fig 4:**
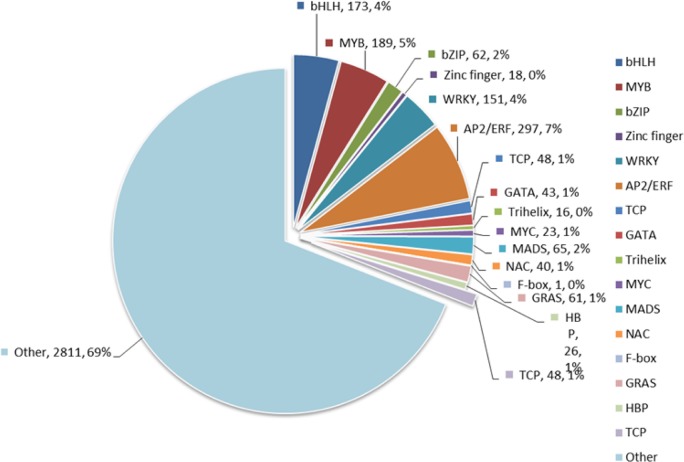
Number of unique transcripts that were annotated as transcription factors.

**Fig 5:**
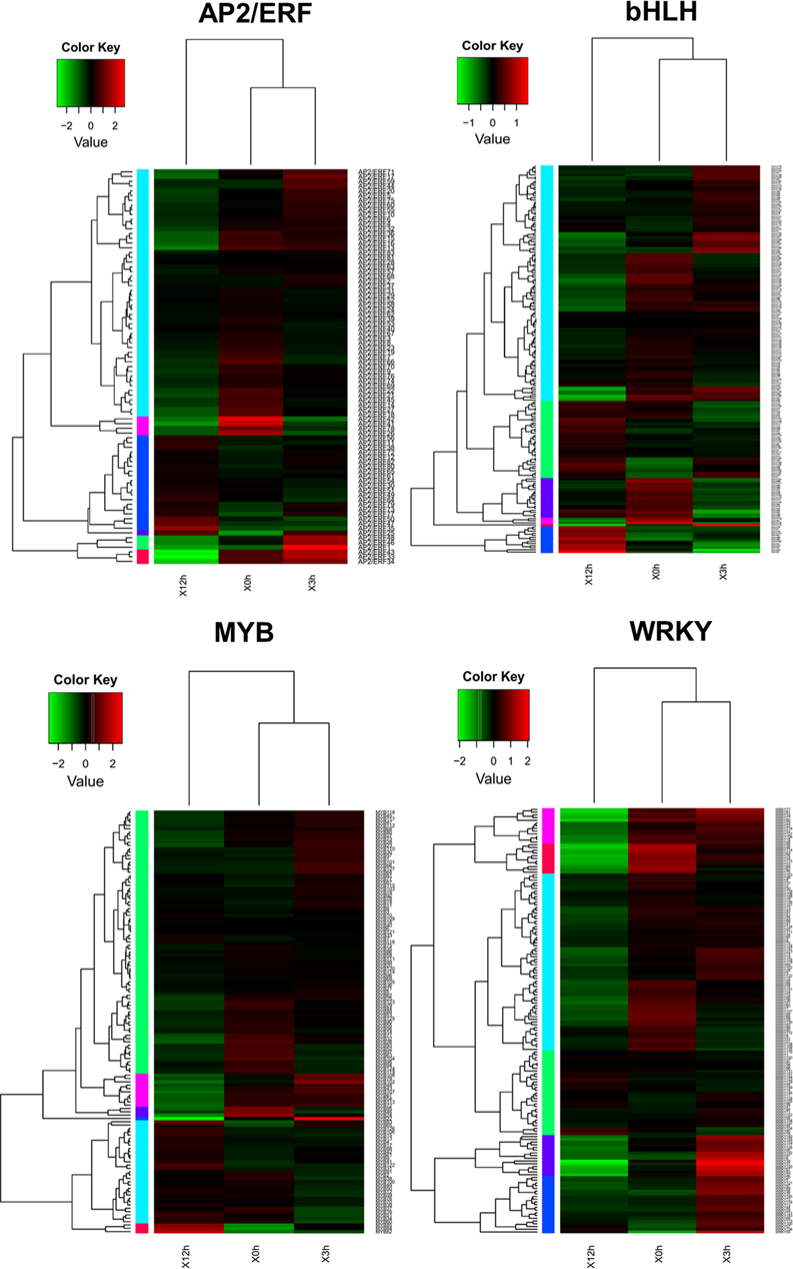
Cluster analysis of the different expression of four important transcription factor families across three comparisons. The differentially expression levels were log10 transformed and are shown with high expression represented by red and low expression represented by blue.

TFs are proteins, binding to specific DNA sequences in the promoters to control and regulate the transcription of genes, which are essential for plants to respond to various stresses and pathogen attacks from environment, such as drought, high salt content and high temperature. In this study, 4072 TFs were identified and classified into 17 different common families by searching from unique transcriptoms (Fig 4). AP2/ERF family was the largest group (297, 7%), followed by MYB (189, 5%), bHLH (173, 4%) and WRKY (151, 4%). This result is similar to the wheat transcriptome, whose largest group is bZIP, followed by MBF1, WRKY and MYB [24], and similar to the result from the Tibetan *Sophora* transcriptome, whose largest group is bZIP, followed by MYB, bHLH, and WRKY [25]. Despite a minor difference, these results further suggest that AP2/ERF, MYB, bHLH, and WRKY TFs are superfamilies in plants, and the expression of most numbers are affected by abiotic and abiotic stress such as heat, low temperature, MeJA and ethylene. To understand the impact of MeJA on these important transcription factors, expression change comparison was studied (Fig 5 and S2 Table) and the result was concluded in Table 1. Notably, two families (bHLH and WRKY) were susceptible to MeJA, including 46 (24.7%) and 68 (43.6%) significantly regulated genes respectively.

**Table 1:** The impact of MeJA on four transcription factor families.

Family name	Up-regulated genes	Down-regulated genes	Up/Down-regulated genes	Untested genes	Significantly regulated genes
**MYB**	21	28	45	33	24
**AP2/ERF**	9	26	22	26	28
**bHLH**	28	65	60	33	46
**WRKY**	15	51	64	26	68

**Fig 6:**
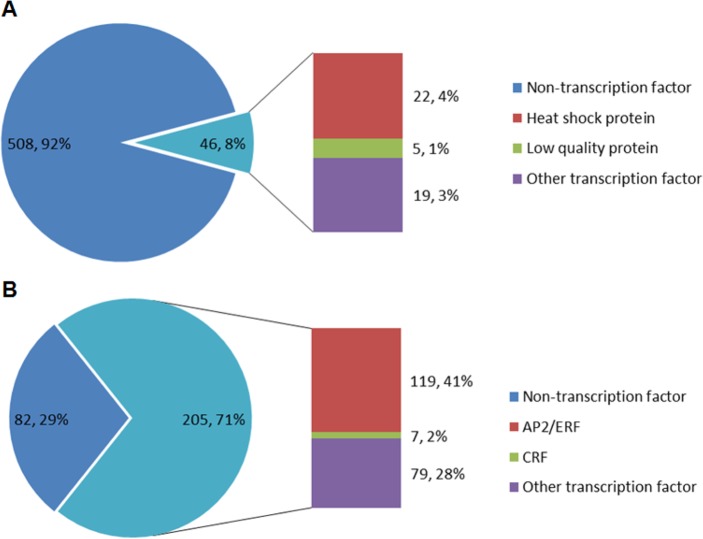
Number of unigenes that were annotated as response gene to hot stress and ethylene. A) Hot-responsive genes. B) Ethylene-responsive genes.

Furthermore, to understand the influence of other stresses from environment, the heat responsive and ethylene responsive proteins were identified through the Nr database. For heat, 554 protein sequences and 46 transcription factors were identified. The result showed that the heat shock protein was the largest group (22, 4%) (Fig 6A). For ethylene, 287 proteins and 205 transcription factors were identified, which contained the largest AP2/ERF family (119, 41%) (Fig 6B).

### 2.5 Verification of expression profile by qPCR

**Fig 7:**
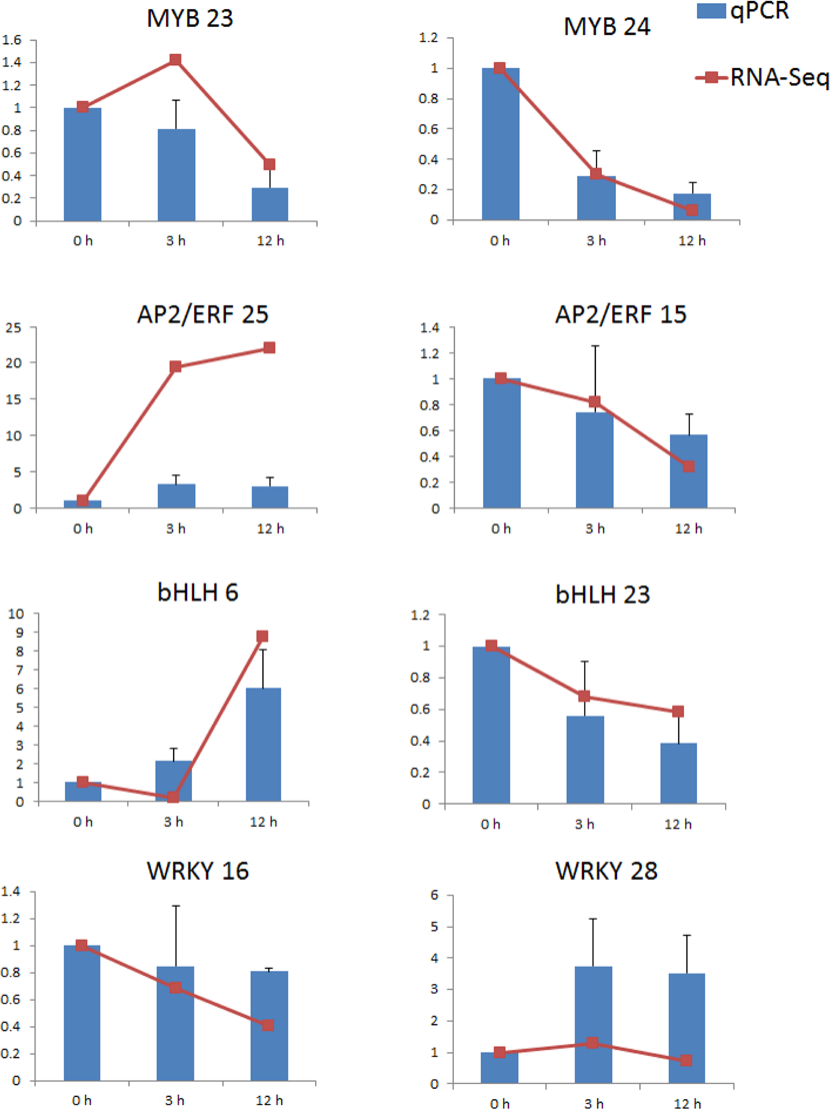
Verification of 8 putative transcription factors from four transcription factor families abovementioned that were involved in the MeJA response by qRT-PCR. The X-axis shows the different time points, and the Y-axis represents the expression levels relative to control levels.

To confirm the gene expression data, 8 transcription factors from 4 crucial transcription factor families abovementioned (AP2/ERF, MYB, bHLH, and WRKY) whose expression was up- or down regulated after MeJA treatment were randomly chosen for qRT-PCR analysis. As shown in Fig 4, the unigene expression trends were similar in both sequencing and transcript data (Fig 7).

### 2.6 Analysis of differential gene expression after treatment of MeJA

**Fig 8:**
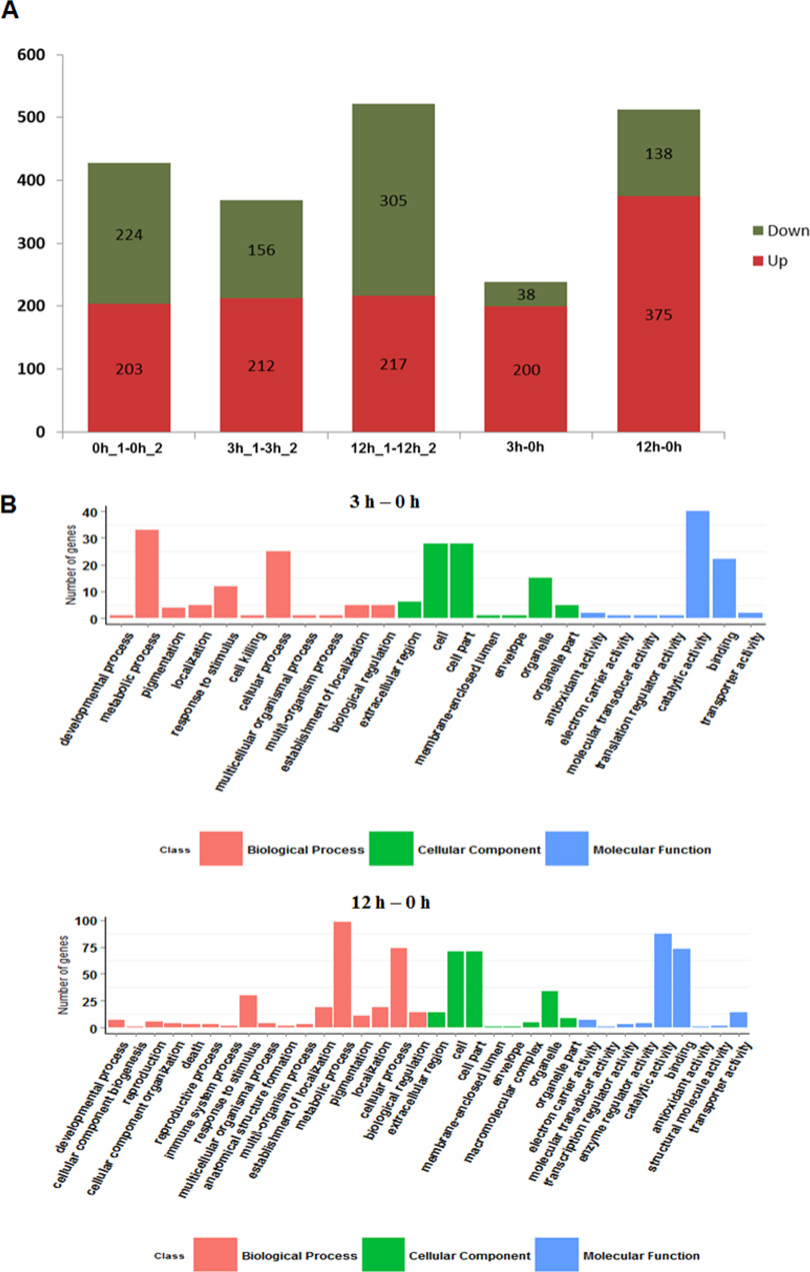
Analyses of unigenes that were of different expression. A) The number of differently expressed genes. High expression is represented by red and low expression represented by blue. The first three columns indicate comparison of two repeats (0 h_1–0 h_2, 3 h_1–3 h_2 and 12 h_1–12 h_2). The last two columns (3 h-0 h and 12 h-0 h) refer to comparison of two different time points. B) Differently expressed genes were categorized by comparison with the GO databases.

After calculating the expression level of each mapped unigene (adjusted p-value < = 0.05), a total of 238 (200 up-regulated genes and 38 down-regulated genes) and 513 unigenes (375 up-regulated genes and 138 down-regulated genes) were induced significantly (>2 folds between the MeJA-treated and control) after 3 and 12 hours of MeJA treatment respectively. Fig 8A showed that the number of different expression gene between two repetitions of 0 h (0 h_1–0 h_2), 3 h (3 h_1–3 h_2) and 12 h (12 h_1–12 h_2) was close, however 3 hours after MeJA treatment (3 h–0 h) showed a decreased number of up-regulated genes, which meant that induction occurred. Interestingly, the up-regulated genes did not increase dramatically until 12 h after MeJA treatment (12 h–0 h), which might indicate that the effectiveness of induction reached a high level after 12 h of MeJA treatment.

To identify the genes that were differentially expressed under MeJA, a categorization was carried out by GO analysis (Fig 8B). By comparing MeJA treatment versus control, the differentially expressed genes abovementioned were functionally assigned to the relevant terms in three categories (‘Biological Process’, ‘Cellular Component’, and ‘Molecular Function’) of the GO database. The GO terms of ‘Cellular Component’ and ‘Molecular Function’ showed a similarity of distribution of differentially expressed genes in both two comparisons (‘cell’ and ‘cell part’ in Cellular Component, ‘catalytic activity’ and ‘binding’ in Molecular Function contained the largest members). However, the type of differentially expressed genes at 12 h was more than that in 3 h. It is also worth noting that the number of ‘metabolic process’ and ‘cellular process’ at 12 h was more than that at 3 h. These data, overall, suggested that ‘metabolic process’ and ‘cellular process’ were strongly affected, probably because MeJA-induced activities of signal pathway in plant mainly led to re-programming of metabolism and cell activity. Furthermore, 12 h after treatment is the top-effected point of MeJA in *E*. *breviscapus*.

Due to the high medicinal value of flavonoids in *E*. *breviscapus*, we mainly focused on the effect of MeJA to biosynthesis of flavonoids (S3 Table). 30 up-regulated genes and 11 down-regulated genes (>10 folds) at both 3 and 12 h after MeJA treatment was identified. The results told that MeJA showed a positive effect on more genes to biosynthesis of flavonoids.

### 2.7 MeJA-induced changes in the metabolites

**Fig 9:**
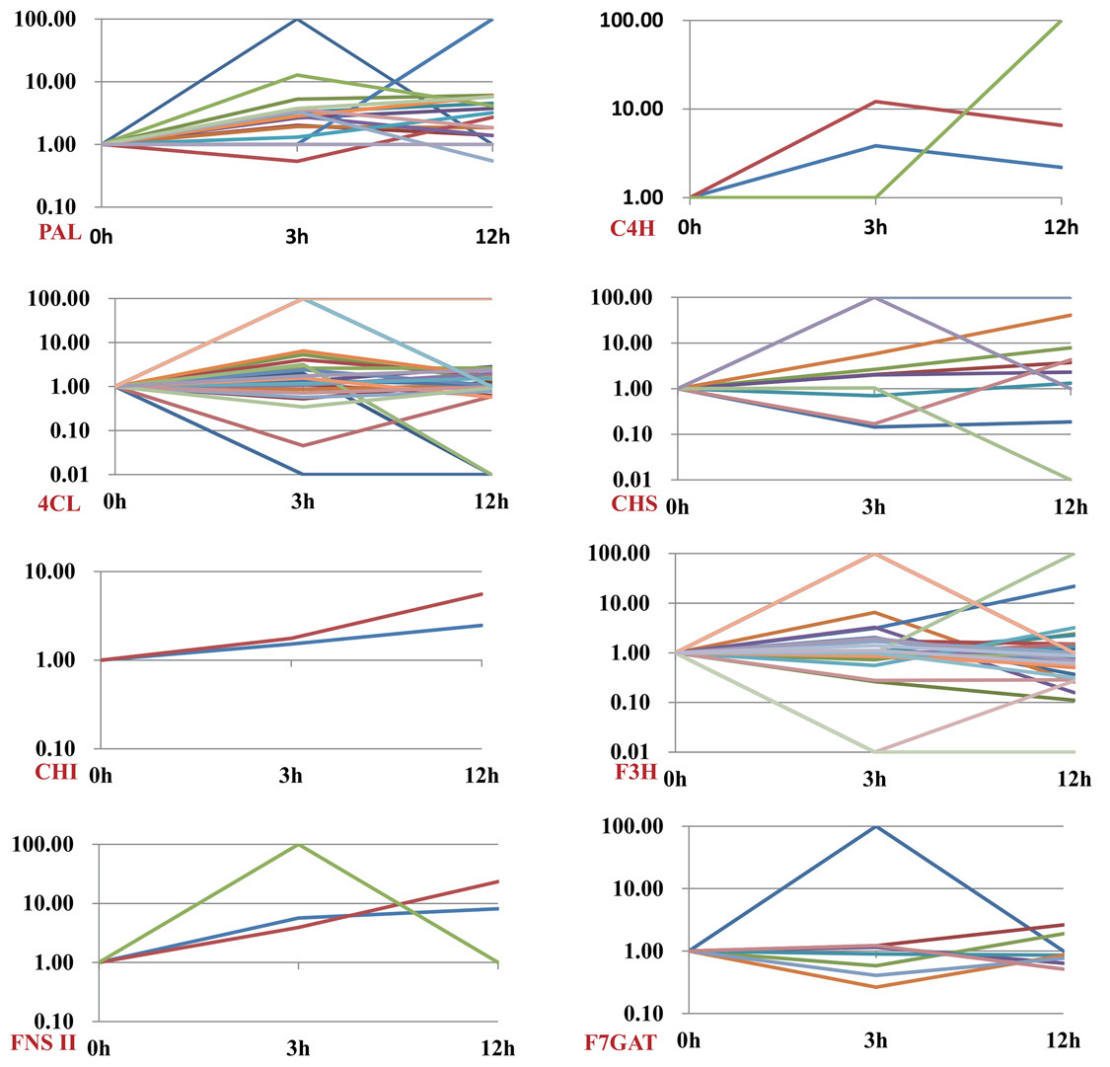
Stimulation of scutellarin and apigenin 7-O-glucoside in *E*. *breviscapus* by MeJA. Content level changed after MeJA treatment. Numerical values indicate the increase in compound levels in MeJA-treated over mock-treated cultures for each day. Three independent biological samples from control and elicitor-treatment plants were analyzed at each time point. **: Significant difference between induced lines to CK, p < 0.01.

Here, we focused on the scutellarin and apigenin 7-O-glucoside, the most important compounds for its medicinal values. To get the richest relative abundance of precursor ions and product ions, the best parameters were chosen (S1 Fig). The spectra of full scan product ion of precursor ions of the two analytes and chromatograms of the standard substance were also listed in S1 Fig The content of scutellarin performed a 2.1-fold increase approximately in day 1 and 2 after MeJA treatment. The content of apigenin 7-O-glucoside increased 3.2 fold in day 2 (Fig 9). Integration of transcripts and metabolites data will be crucial for the study of metabolism in plants, both at the regulatory and catalytic levels. Result indicated that inductive effect of MeJA on compounds’ accumulation had an obvious delay. Compared the variation trend of genes’ expression (S2 Fig) and compounds’ accumulation (Fig 9), the genes showed similar variation trend with compounds and higher expression level (> 10 fold) may more like strongly catalyze the metabolism of scutellarin, such as *PAL1*, *PAL9*, *C4H2*, *4CL29*, *CHS10*, *F3H30*, *FNS II3*, *F7GAT1*.

### 2.8 Co-expression analyses

**Fig 10:**
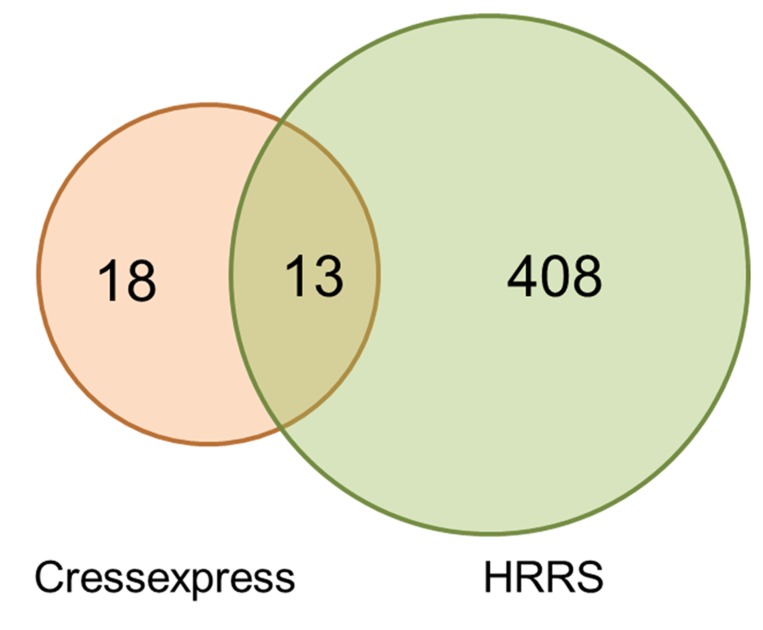
Co-expression analysis of known flavonoids biosynthetic genes. Two different co-expression analyses were performed using CressExpress [23] and a high-resolution root spatio-temporal (HRRS) expression dataset [24]. In each of these *PAL2* (AT3G53260), *PAL4* (AT3G10340), *C4H* (AT2G30490), *4CL1* (AT1G51680), *4CL2* (AT3G21240), *4CL3* (AT1G65060), *4CL5* (AT3G21230), *CHS* (AT5G13930), *CHI* (AT3G55120), *FNS I* (AT5G08640) and *FNS II* (AT5G63580) were used as bait. Candidate genes had to be co-expressed with two or more flavonoids biosynthesis genes in order to be retained on the co-expressed gene list. 13 genes were found to be co-expressed in all two analyses. Gene identities are given in S5 Table.

Here, we predicted more candidates for involvement in the biosynthesis of flavonoids, based on analyses designed to identify genes co-expressed with known components of the flavonoids biosynthetic pathway (Fig 10). Of the 13 genes in *E*. *breviscapus* and 9 genes in *Arabidopsis thaliana* identified by each of two different co-expression software tools, publicly available datasets and our datasets, 2 were certificated genes to regulate the flavonoids metabolism in *A*. *thaliana* (*AT5G48930* and *AT5G44110*), and others also showed an indirect interaction with flavonoids metabolism, such as lignin biosynthetic process (*AT5G13420*), coumarin biosynthetic process (*AT4G23850*). However the 13 predicted genes in *E*. *breviscapus* had no known role in the biosynthesis of flavonoids (S4 Table).

## Materials and Methods

### 3.1 Plant material

The seeds of *E*. *breviscapus* were purchased from Southwest Forestry University (102.75° E, 25.06° N). The plant was grown under normal glasshouse conditions and identified by Professor Hanming Zhang, who is a famous pharmacognosy researcher in China (School of Pharmacy, Second Military Medical University, Shanghai, China,).

To elucidate the mechanism of MeJA response, 90-day-old *E*. *breviscapus* plants (6 leaves approximately) in pots were randomly selected and assigned to one of three different groups (2 plants each group): (1) control plants treated with 0.1% acetone, (2) plants treated with MeJA (1.5 Mm) and harvested at 3 h, (3) plants treated with MeJA (1.5 Mm) and harvested at 12 h. MeJA was dissolved in 0.1% acetone for a concentration of 1.5 mM. Different solutions (15 mL) were evenly sprinkled on every leaf of the plants in different groups.

### 3.2 Total RNA isolation, RNA-seq library construction and sequencing

The leaves from the middle position of the plants were collected from the three groups and then placed in liquid nitrogen immediately. Total RNAs were extracted using TRIzol+ Reagent (TianGen) according to the manufacturer’s instruction. An equal quantity of RNA from all plants was blended for cDNA library construction to obtain the transcriptome data. A normalized cDNA library was constructed with 1 μg of total RNA. The sequencing libraries were generated using NEBNext Ultra Directional RNA Library Prep Kit for Illumina (NEB, USA) with the manufacturer’s instruction, which were sequenced on an Illumina HiSeq 2000 platform for paired-end reads. All sequencings were purchased from the Suzhou Genewiz Bio-pharm Technology Corporation (Jiangsu, China).

### 3.3 De novo assembly and functional annotation

For high-quality clean data, the raw sequence data was purified by trimming adapter sequences and removing low-quality sequences. The clean reads were assembled using Trinity software as described for assembly without a reference genome (http://trinityrnaseq.sourceforge.net). After assembling, BLASTx alignment (*e* value < 1.00E-5) of all the unigenes were annotated against protein databases, including the NCBI non-redundant (Nr) protein database, Gene Ontology database (GO), Swiss-Prot protein database, Kyoto Encyclopedia of Genes and Genomes (KEGG) pathway database, and the Cluster of Orthologous Groups (COG) database.

### 3.4 Validation by quantitative real-time PCR

To confirm the gene expression data, 8 unigenes (MYB23, MYB24, AP2/ERF25, AP2/ERF15, bHLH6, bHLH2, WRKY16 and WRKY28) were randomly chosen from four transcription factor abovementioned for qRT-PCR analysis (S4 Table). For the quantitative RT-PCR of the mRNAs, 1 ug of total RNA was reversely transcribed by Superscript III Reverse Transcriptase (Invitrogen, USA). Real-time PCR was performed using SYBR premix Ex Taq (Takara, Japan) following the manufacturer’s instruction. The PCR amplification was performed under the following conditions: 95°C for 30 s, followed by 40 cycles of 95°C for 5 s, 53°C for 10 s and 72°C for 20 s. Three independent biological replicates for each sample and three technical replicates for each biological replicate were analyzed. All primers used were listed in S5 Table.

### 3.5 Co-expression analyses

Analyses to identify genes that were co-expressed with known flavonoids biosynthesis genes were performed using two different tools/datasets; CressExpress and a high-resolution root spatiotemporal (HRRS) dataset. For each analysis, 11 known flavonoids biosynthesis genes were used as query baits (S6 Table). CressExpress, a co-expression analysis tool, can rank co-expressed genes based on their common connections with two or more query genes [26]. Similarly, the HRRS dataset that was analyzed with the graphical representation tool was featured by Brown et al. to identify candidate genes that were spatially co-expressed with each flavonoids biosynthesis gene [27, 28]. The final lists from both the two analyses were compared to reveal common genes identified.

### 3.6 Compounds extraction

Plants were dried at 50°C. The dried sample (2.0 mg) was ground into powder and extracted twice with 70% methanol (1 mL) by sonication for 30 min. The supernatant was diluted with 70% methanol to 2 mL. Before analysis, the extract solution should be filtered through a 0.2 μm organic membrane.

### 3.7 Compound analysis

All samples were carried on an Agilent 1200 series HPLC and interfaced to an Agilent 6410 triple-quadrupole mass spectrometer equipped with an electrospray ionization source (Agilent Corporation, MA, USA). A ZORBAX SB-C18 column (3.5 μm, 2.1×150 mm, I.D. Agilent Corporation, MA, USA) and a C18 guard column (5 μm, 4.0×2.0 mm, Agilent Corporation, MA, USA) was used. Acetonitrile-5 mM ammonium acetate solution (the concentration of acetonitrile remain 30% in 2.0 min, v/v) was used as mobile phase for the gradient elution at a flow rate of 0.3 ml/min. The column temperature was maintained at 30°C. The injection volume was 10 μL and the analysis time was 2.0 per sample. The ESI source in negative mode was chosen.

## Conclusions

In this study, we used high-throughput sequencing data to reveal transcriptome re-programming of *E*. *breviscapus* upon MeJA elicitation. A large number of candidate genes involved in MeJA response were identified. Furthermore, using two different co-expression analyses, we predicted 13 candidate genes involved in flavonoids biosynthesis. This work represents a fully characterized transcriptome and provides a valuable resource for genetic and genomic studies. In addition, these results will be helpful for further metabolic engineering to increase the contents of the active compounds.

## Supporting Information

S1 Fig A liquid chromatographic-tandem mass spectrometric method for the quantitation of two compounds involved in scutellarin pathway. **A)** Optimized MRM parameters for scutellarin and apigenin 7-O-glucoside. B) Mass spectrum, product ion spectrum and MRM chromatograms of two compounds.
(EPS)

S2 Fig Variation tendency of biosynthetic genes in scutellarin pathway. (EPS)

S1 Table Summary of the GO, COG and KEGG classifications of assembled unigenes. (XLSX)

S2 Table The list of genes of four transcription factor families. (XLSX)

S3 Table The effect of MeJA to biosynthesis of flavonoids. (XLSX)

S4 Table The list of co-expression genes in *E*. *breviscapus* and *A*. *thaliana*. (XLSX)

S5 Table Primers used in this experiment. (DOC)

S6 Table The list of known flavonoids biosynthesis genes were used as query baits in analyses of co-expression. (XLSX)
