# Supplementary material for: Deep Sequencing Reveals the Effect of MeJA on Scutellarin Biosynthesis in Erigeron breviscapus
Source: PLoS One. 2015 Dec 14;10(12):e0143881. doi: 10.1371/journal.pone.0143881 (PMC4687647; doi:10.1371/journal.pone.0143881)
Supplement: S5 Table — (DOC) [file pone.0143881.s007.doc]

Primers used in this experiment

| primers for qRT-PCR | |
| --- | --- |
| Name | Sequences 5’-3’ |
| MYB23-F | TCCTTCCAACCACCATTATCACA |
| MYB23-R | TGAGATGATGAATGTGGCACATTAT |
| MYB24-F | TGAAAATCATTTTCAAACCGTCGCC |
| MYB24-R | CCCTGGGAGTCCTAATGACAATGTA |
| AP2/ERF25-F | CCCAAGCCAGTTTCTACATCTCC |
| AP2/ERF25-R | CAAGGTCGACATAAAGATCCGGT |
| AP2/ERF15-F | TACACAGATGATCAAACCACAACCA |
| AP2/ERF15-R | TTGAAAGTACCGAGCCAAACTCTT |
| bHLH6-F | TGTCTAGCAGAAGATCTCGCTCTCA |
| bHLH6-R | TTAAGTCCTCGACTTCTCTGTGCAA |
| bHLH23-F | GTGAATAGAATGAACATCTCGCCG |
| bHLH23-R | CCAGGATGGCATATGCATGAAA |
| WRKY16-F | TTAGTTCGATGATGGCTAATCACG |
| WRKY16-R | CCCTTAAGCCGAGATCCTTCAA |
| WRKY28-F | AACATACTGTTAGACAACCCCACCA |
| WRKY28-R | TGCTGGTGATGTTGTTGCAAAA |
| UBQ-F | ACCCTCACGGGGAAGACCATC |
| UBQ-R | ACCACGGAGACGGAGGACAAG |
